# MiRNA-Sequence Indicates That Mesenchymal Stem Cells and Exosomes Have Similar Mechanism to Enhance Cardiac Repair

**DOI:** 10.1155/2017/4150705

**Published:** 2017-01-22

**Authors:** Lianbo Shao, Yu Zhang, Beibei Lan, Juanjuan Wang, Zhiwei Zhang, Lulu Zhang, Pengli Xiao, Qingyou Meng, Yong-jian Geng, Xi-yong Yu, Yangxin Li

**Affiliations:** ^1^Department of Cardiovascular Surgery, The First Affiliated Hospital and The Institute for Cardiovascular Science, Soochow University, Suzhou, Jiangsu 215123, China; ^2^Department of Internal Medicine, University of Texas, Houston, TX 77583, USA; ^3^Guangzhou Medical University, Guangzhou, Guangdong 510080, China

## Abstract

Mesenchymal stem cells (MSCs) repair infarcted heart through paracrine mechanism. We sought to compare the effectiveness of MSCs and MSC-derived exosomes (MSC-Exo) in repairing infarcted hearts and to identify how MSC-Exo mediated cardiac repair is regulated. In a rat myocardial infarction model, we found that MSC-Exo inhibited cardiac fibrosis, inflammation, and improved cardiac function. The beneficial effects of MSC-Exo were significantly superior compared to that of MSCs. To explore the potential mechanisms underlying MSC-Exo's effects, we performed several in vitro experiments and miRNA-sequence analysis. MSC-Exo stimulated cardiomyocyte H9C2 cell proliferation, inhibited apoptosis induced by H_2_O_2_, and inhibited TGF-*β* induced transformation of fibroblast cell into myofibroblast. Importantly, novel miRNA sequencing results indicated that MSC-Exo and MSCs have similar miRNA expression profile, which could be one of the reasons that MSC-Exo can replace MSCs for cardiac repair. In addition, the expression of several miRNAs from MSC-Exo was significantly different from that of MSCs, which may explain why MSC-Exo has better therapeutic effect than MSCs. In conclusion, this study demonstrates that MSC-Exo could be used alone to promote cardiac repair and are superior to MSCs in repairing injured myocardium.

## 1. Introduction

Mesenchymal stem cells (MSCs) have been shown to repair infarcted myocardium by secreting paracrine factors [1–3]. Several studies have demonstrated the safety and efficacy of MSCs in clinical trials [4–7]. However, cell transplantation-related contamination, cell death, and immune rejection are the major concerns in clinical practice. Therefore, alternative treatment strategies, such as exosome-based therapy, are preferred [8, 9].

Exosomes are secreted nanosized membrane vesicles with diameters within 30–100 nm. Exosomes contain functional proteins, mRNAs, microRNAs (miRNA) [10], and tRNA species [11] that play important roles in intercellular communication [12, 13]. MSCs derived exosomes (MSC-Exo) can improve heart function after infarction [14–17]. No studies have directly compared the effects of MSC-Exo and MSCs; therefore, it is not known whether MSC-Exo could replace MSCs for cardiac repair. The molecular mechanisms responsible for MSC-Exo and MSCs mediated cardiac repair are also unknown.

In this study, we compared the effects of MSC-Exo and MSCs in a rat myocardial infarction model and performed in vitro experiments to determine the impacts of MSC-Exo on cell proliferation, apoptosis, and myofibroblast formation. We also performed novel miRNA sequencing to compare the miRNA expression profile between MSC-Exo and MSCs.

## 2. Materials and Methods

### 2.1. Ethics Statement

All animals were obtained from the Experimental Animal Center of Soochow University (Suzhou, China). Animal experiments were approved by the Institutional Animal Care and Use Committee of Soochow University. All the procedures were in compliance with the Guide for the Care and Use of Laboratory Animals, published by the US National Institutes of Health (NIH Publication number 85-23, revised 1996).

### 2.2. Isolation and Culture of MSCs

Bone marrow MSCs were isolated from male Sprague-Dawley (SD) rats as previously described [18]. Briefly, bone marrow cells were flushed out with basic culture medium from the femur and tibia bones and seeded in culture dishes containing Dulbecco's modified Eagle's medium : nutrient mixture F-12 (DMEM : F12, HyClone), supplemented by 10% fetal bovine serum (FBS) (Biological Industries) and penicillin (100 U/mL)/streptomycin (100 *μ*g/mL) (Sigma). Cells were incubated at 37°C in a humidified atmosphere containing 5% CO_2_. After 48 hours, the dishes were washed with fresh culture medium to remove the nonadherent cells. Cells were harvested with 0.25% trypsin-EDTA (Sigma) and passaged at 5 × 10^3^ cells/cm^2^. The MSCs (passage 3 [P3]) were identified by flow cytometry using antibodies against CD90-APC (Allophycocyanin, BD Pharmingen), CD45-PE-Cy7 (Phycoerythrin-Cy7, BD Pharmingen), and CD11b-FITC (Fluorescin isothiocyanate, Biolegend). MSCs at P3, P4, and P5 were used for the experiments.

### 2.3. Harvest and Identification of MSC-Exo

Exosomes were harvested from MSCs at passage 4. MSCs were cultured in DMEM : F12 containing 10% FBS. The FBS has been centrifuged at 100,000*g* in order to eliminate preexisting bovine-derived exosomes [19]. After 48 hours, exosomes derived from MSC culture supernatants were isolated using total exosome isolation kit (Life Technology), which yields high quantities of purified exosomes. The culture media collected from MSCs were centrifuged at 2,000*g* for 30 minutes to remove dead cells and debris, and then the media were transferred to a new tube, containing 0.5 volumes of the Total Exosome Isolation reagent. The mixture was incubated at 4°C overnight and centrifuged at 10,000*g* for 1 hour at 4°C. The pellets were resuspended in PBS and stored at −80°C. Protein concentration of MSC-Exo was determined using a BCA protein assay kit (Takara) [20]. The morphology of MSC-Exo was identified by transmission electron microscope, and the phenotype of MSC-Exo was analyzed by flow cytometry. Because exosomes are too small to be captured directly by flow cytometry, MSC-Exo was prebound to aldehyde/sulfate latex beads (4 *μ*m; Molecular Probes; Invitrogen) to amplify channel signal and then incubated with a PE conjugated antibody against CD63 (Abcam) which is a specific marker for exosome [21]. Furthermore, CD63 protein was detected by Western blot using a specific antibody against CD63 (Abcam).

### 2.4. MI Induction and Implantation of MSCs and MSC-Exo

To assess the effects of MSC-Exo in acute myocardial infarction (MI), MI was induced in 30 male SD rats (260–280 g) as previously reported [22–25]. Animals were anesthetized with intraperitoneal injection of 80 mg/kg pentobarbital, and then the fur of the neck and chest areas were shaved. A chest retractor was positioned within the fourth intercostal space. After the left ventricle was exposed, the left coronary anterior descending coronary artery was ligated with an 8-0 nylon suture. Successful induction of MI was verified by color change immediately in the infarcted area. After LAD was ligated, PBS (20 *μ*L), MSCs (1 × 10^6^ cells), and MSC-Exo (20 *μ*g/20 *μ*L) were injected into two different sites along the infarct border region. In sham operated control rats, the procedure was identical, except that the LAD was not ligated. Penicillin (1.5 × 10^5^ U/mL) was delivered (i.p) after the surgery.

### 2.5. Cardiac Function

Under anesthesia (1.0% inhaled isoflurane), echocardiography was performed to evaluate cardiac function at baseline and 1 and 7 days after MI using a 21 MHz transducer (VisualSonics). The left ventricular ejection fraction (LVEF) and fraction shorting (FS) were calculated as previously described [26]. All procedures and analysis were performed by a researcher who was blinded to treatment groups.

### 2.6. Statistical Analysis

Data were presented as mean ± SD. Statistical significance between 2 groups was determined by nonparametric test (Mann–Whitney test). Comparisons among multiple groups were analyzed by Kruskal–Wallis test, with Dunn's posttest to compare all pairs of groups. *P* < 0.05 was considered to indicate significant difference.

## 3. Results

### 3.1. Identification and Characterization of MSC and MSC-Exo

**Figure 1:**
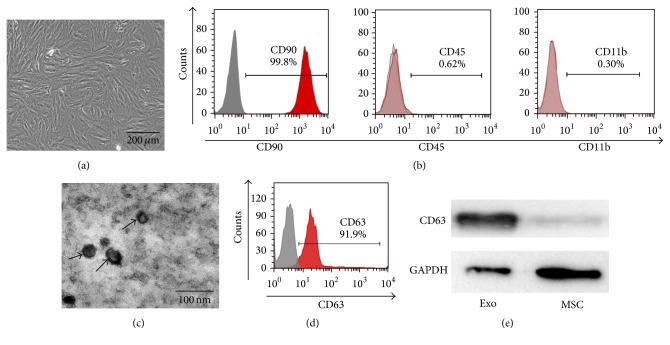
Characterization of MSCs and MSC-Exo. (a) The morphology of MSCs was observed under microscope; scale bar = 200 *μ*m. (b) Flow-cytometric analyses showed that cultured MSCs from rats were positive for CD90 and negative for CD45 and CD11b. (c) The morphology of MSC-Exo was observed under an electron microscope. Bar = 100 nm. (d) The expression of exosome marker CD63 was identified by flow-cytometric analyses. (e) Western blot analysis of CD63 protein in MSC-Exo.

Rat bone marrow MSCs were plastic adherent and spindle shaped under a light microscope (Figure 1(a)). Flow cytometry analysis showed that MSCs were positive for CD90, but negative for CD45 and CD11b (Figure 1(b)), which was consistent with previous report [27]. MSC-Exo appeared as small round particles with typical cup-shaped morphology, as revealed by electron microscopy (Figure 1(c)). Flow cytometry analysis showed that MSC-Exo expressed high levels of CD63 (Figure 1(d)), and the results were confirmed by Western blot (Figure 1(e)).

### 3.2. MSC-Exo Treatment Preserves Myocardial Function after MI

**Figure 2:**
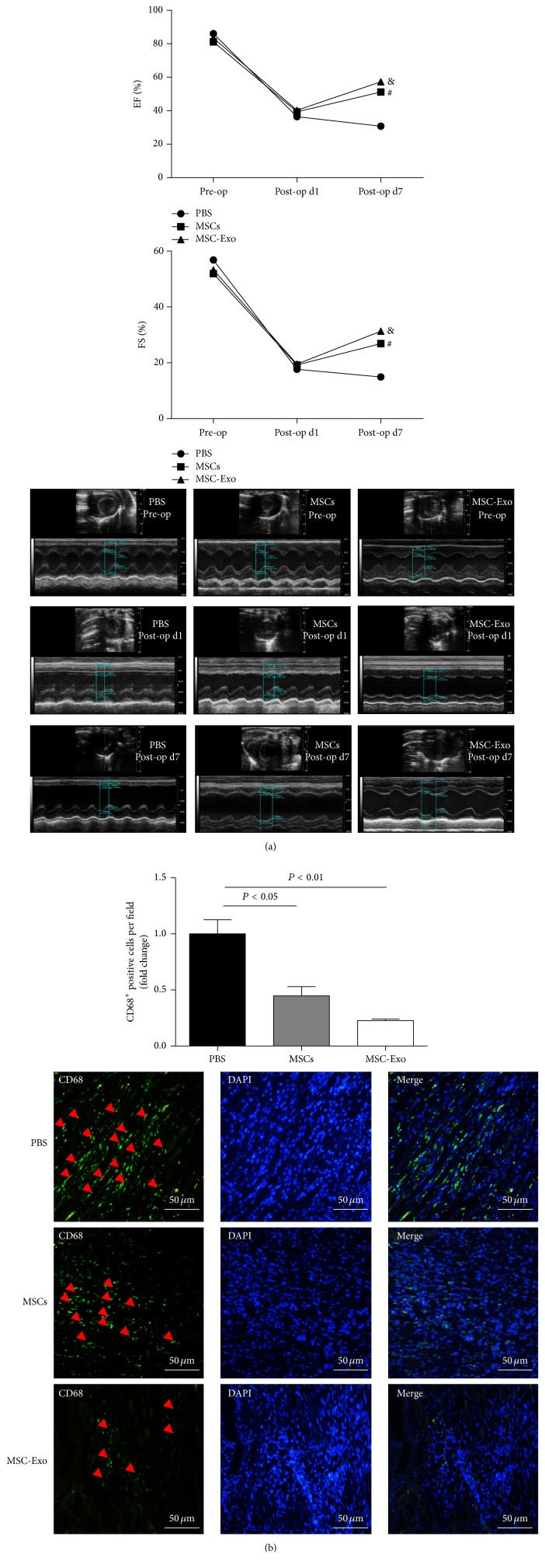
Analysis of rat myocardial function and inflammation after MSC-Exo and MSCs transplantation. (a) Representative echocardiography images of left ventricular ejection fraction (LVEF) and fraction shorting (FS) in the PBS, MSCs, and MSC-Exo-injected groups. LVEF and FS were measured preoperatively and at 1 and 7 days post-MI induction (*n* = 5/group). (b) MSC-Exo reduces inflammation in the peri-infarct myocardium. PBS control, MSCs, and MSC-Exo were injected into the peri-infarct zones and heart samples were harvested 1 week after injection. Heart sections were stained with anti-CD68 antibody (green) to detect inflammation in the peri-infarct zone. Bar = 50 *μ*m. # represents MSCs group versus PBS group, *P* < 0.05; & represents MSC-Exo group versus PBS group, *P* < 0.05.

We evaluated the therapeutic effects of MSC-Exo and MSCs in a myocardial infarction (MI) model. Echocardiography was performed at baseline and 1 and 7 days after the MI surgery. As shown in Figure 2(a), LVEF was not significantly different among the groups preoperatively and 1 day after surgery. However, 7 days after MI surgery, the LVEF was markedly increased in the MSC-Exo group and MSCs groups compared with the control group (Figure 2(a)) (MSC-Exo group: 59.66 ± 4.38%; MSC group: 50.23 ± 3.45%; control: 30.77 ± 4.13%. MSC-Exo versus control, *P* < 0.05)

The FS was markedly increased in the MSC-Exo group and MSCs groups compared with the control group (Figure 2(a)) (MSC-Exo group: 31.31 ± 7.03%; MSC group: 26.87 ± 2.67%; control: 14.93 ± 2.31%. MSC-Exo versus control, *P* < 0.05).

### 3.3. MSC-Exo Treatment Reduces Fibrosis

Masson trichrome staining was performed 1 week after MSC-Exo injection in order to assess the size of MI. As shown in Supplemental Figure  1(a) (see Supplementary Material available online at https://doi.org/10.1155/2017/4150705), the blue color represents fibrotic tissue and the red color shows normal myocardium. Through quantitative analyses using Image J software, we found that the percentage of fibrotic area in the entire cross-sectional area and the percentage of fibrosis length in the entire internal circumference were both significantly reduced in the MSC-Exo group compared with MI group (Supplemental Figures  1(b) and 1(c)).

### 3.4. MSC-Exo Treatment Reduces Inflammation

To determine whether MSC-Exo treatment could reduce inflammation, PBS, MSCs, and MSC-Exo were injected into the peri-infarct zones. One week after myocardial infarction induction, hearts sections were stained with anti-CD68 antibody to identify infiltrated inflammatory cells in infarcted myocardium. Our data showed that CD68 expression in the MSCs group was significantly decreased compared with PBS group (*P* < 0.05). Importantly, the difference in CD68 expression between the MSC-Exo group and PBS group is highly significant (*P* < 0.01) (Figure 2(b)).

### 3.5. MSC-Exo Are Internalized by H9C2 and BJ Cells

MSC-Exo was labeled with the fluorescent dye PKH26 in order to determine whether MSC-Exo could be internalized into cardiomyocyte H9C2 and fibroblast BJ cells. After incubating the labeled MSC-Exo with H9C2 or BJ cells for 12 hours, internalization of MSC-Exo was examined under a fluorescent microscope (Supplemental Figure  2). The red fluorescence observed in the cytoplasm of H9C2 and BJ cells indicated that PKH26-labeled MSC-Exo have been successfully internalized by H9C2 and BJ cells.

### 3.6. MSC-Exo Promotes Proliferation and Inhibits Apoptosis of H9C2 Cells

In order to assess the effect of MSC-Exo on cell proliferation, H9C2 cells were incubated with different amount of MSC-Exo for 48 hours and proliferation was determined by Edu assay. As shown in Supplemental Figures  3(a) and 3(b), the proliferative capacity of H9C2 cells was significantly enhanced by MSC-Exo in a dose-dependent manner.

It was shown that MSCs could inhibit H9C2 apoptosis via paracrine mechanisms [15]. Using the Annexin V/PI assay, we found that H9C2 cells were resistant to H_2_O_2 _induced apoptosis after treatment with MSC-Exo in a dose-dependent manner (Supplemental Figure  4), suggesting that the paracrine effect exerted by MSCs may be mediated by MSC-Exo.

### 3.7. MSC-Exo Inhibits Fibroblast Transformation

It is known that TGF-*β* promote cardiac fibrosis by transforming fibroblasts into myofibroblasts [28]. We evaluated the impact of MSC-Exo on the expression of *α*-SMA, a defining marker of myofibroblast. Our data demonstrate that TGF-*β* induced expression of *α*-SMA in BJ fibroblast cells was reduced by MSC-Exo dose dependently (Supplemental Figure  5).

### 3.8. Sequencing Results of miRNA from MSC-Exo and MSC

**Figure 3:**
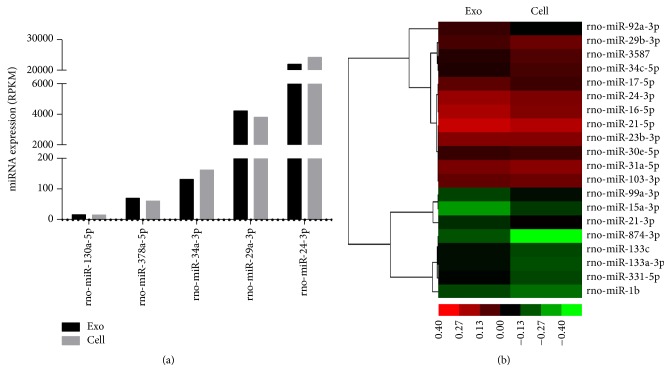
(a) miRNA expression profiling. Total RNA was extracted from MSC-Exo and MSCs using Qiagen miRNeasy Mini Kit. The sequence was detected by HiSeq 2500 platform. The RPKM stands for the miRNA expression. RPKM: read per kilobases per millionreads. (b) Heat map of miRNA sequencing data from MSC-Exo and MSCs. Green: downregulated. Red: upregulated.

Sequencing analysis of miRNA from MSC-Exo and MSCs was performed by the HiSeq 2500 platform as described in the supplemental methods. We found that MSC-Exo and MSCs had similar miRNA sequence profile in general, as quantified by read per kilobases per millionreads (RPKM). Based on the literature, we focused on the miRNAs which play critical roles in cardiac function. We found that the expression of miR-130, miR-378, and miR-34, which negatively regulate cardiac functions, was relatively low. The expression of miR-29 and miR-24, which positively regulate cardiac functions, was relatively high (Figure 3(a)).

**Figure 4:**
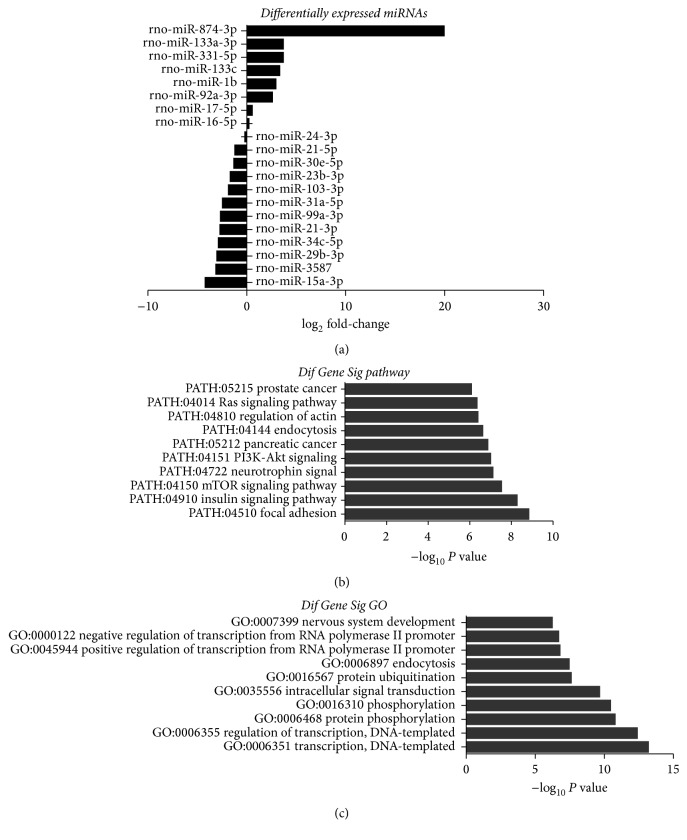
Differentially expressed miRNAs, pathway analysis, and gene ontology (GO) in MSC-Exo compared with MSCs. (a) List of the differentially expressed miRNAs and the log_2_ fold-changes are indicated. (b) Pathways associated with increased expression of miRNAs in MSC-Exo. The vertical axis is the pathway category and the horizontal axis is the enrichment of pathways. (c) GO category associated with increased expression of miRNAs in MSC-Exo. The vertical axis is the GO category, and the horizontal axis is the enrichment of GO.

To further characterize the difference of miRNA expression between MSC-Exo and MSCs, the hierarchical cluster of miRNA expression was made, which indicated that the expression of several miRNAs (including miR-15) derived from MSC-Exo was significantly different from that of MSCs (Figure 3(b)). Differentially expressed miRNAs were also defined by using a FDR (False Discovery Rate) threshold and log_2_ FC (fold-change) analysis through EBSeq algorithm. The threshold of truly significant miRNA was defined as FDR < 0.05 and log_2_ FC > 1 or <−1. For example, the expression of miR-21 and miR-15, which regulate cardiac functions, was significantly lower in MSC-Exo compared to that in MSCs (Figure 4(a)). The target gene of miRNA was predicted with TargetScan and Miranda software. The predicted targets of the differentially expressed miRNAs were then analyzed in terms of their gene ontology (GO) categories and pathways using Fisher's exact test and *χ*^2^ test. GO analysis was applied to analyze the main function of the differentially expressed genes according to the GO which is the key functional classification of NCBI. Pathway annotations of genes were predicted from KEGG (http://www.genome.jp/kegg/). We found several pathways, including PATH: 04151 (PI3k-Akt pathway) and PATH: 04150 (mTOR pathway) (Figures 4(b) and 4(c)), which were significantly upregulated in MSC-Exo compared to that in MSCs.

## 4. Discussion

In the present study, we demonstrate that MSC-Exo reduced inflammation, inhibited fibrosis, and improved cardiac function in a rat myocardial infarction model. The effects of MSC-Exo were significantly superior to that of MSCs. Importantly, the novel miRNA sequencing results indicated that MSC-Exo and MSCs had similar miRNA expression profile, which is one of the reasons why MSC-Exo can replace MSCs to treat myocardial infarction. However, miRNA profiling analysis also revealed that the expression of several miRNAs was significantly different between MSC-Exo and MSCs, which may explain why MSC-Exo are superior to MSCs in treating MI. To our knowledge, these novel findings have not been reported before.

Furthermore, one of the primary barriers limiting the effectiveness of stem cell based therapy is the harsh ischemic microenvironment which may kill most of the injected cells before they could engraft and produce any beneficial factors. In this context, MSC-Exo is preferred because this approach is cell free and the injected MSC-Exo could be internalized by nearby cardiac cells and fibroblasts and enhances myocardial function by reprogramming the cardiac cells and fibroblasts.

Our recent study demonstrated that MSC-Exo could stimulate the proliferation and migration of cardiac stem cells (CSCs) in a rat myocardial infarction model [29]. Others suggested that CD34^+^ stem cell derived exosomes promote angiogenesis and inhibit apoptosis and fibrosis [30, 31]. Here, we demonstrated that MSC-Exo prevented the transformation of fibroblast to myofibroblast for the first time. This novel finding explains why MSC-Exo injection could reduce cardiac fibrosis after MI.

MiRNAs, shuttled by exosomes, are among the most important molecular factors controlling cardiac repair [8]. Therefore, we evaluated the miRNA expression profile in MSC-Exo and MSCs through miRNA sequencing. Our results showed high expression of miR-29 and miR-24 in both MSC-Exo and MSCs. Previous studies have shown that enhanced expression of miR-29 prevented kidney fibrosis by reducing the expression of collagen [32]. Upregulation of miR-24 limits aortic vascular inflammation [33]. Importantly, in vivo expression of miR-24 in a mouse MI model inhibited cardiomyocyte apoptosis, attenuated infarct size, and reduced cardiac dysfunction [34]. Moreover, our results showed that the expression of miR-34 was decreased in both MSC-Exo and MSCs. Previous studies showed that inhibition of miR-34 expression in vivo using LNA-based antimiRs or antagomiRs improved cardiomyocyte survival after MI and thereby preserved cardiac contractile function [35, 36].

Moreover, the low expression of miR-130 and miR-378 in both MSC-Exo and MSCs found in our study is in line with other reports that high expression of the miR-130 and miR-378 caused K ion channel dysfunction in cardiac stem cell and cardiac hypertrophy [37, 38]. Therefore, increased expression of miR-29 and miR-24 and reduced expression of miR-34, miR-130 and miR-378 may be responsible for the beneficial effects exerted by MSC-Exo.

Furthermore, our findings that the expression of miR-21 and miR-15 was significantly lower in MSC-Exo compared to MSC, which are in line with previous reports that downregulation of miR-21 prevents hypertrophy [39] and inhibition of miR-15 prevents cardiac ischemic injury [40]. These findings may explain why MSC-Exo had better effects in cardiac repair. Certainly, we need to identify the exact targets of the miRNAs and define the pathways in future studies in order to better understand how these miRNA might affect cardiac repair.

## 5. Conclusion

This is the first study to compare the effects of MSC-Exo and MSCs in a rat acute myocardial infarction and demonstrates that MSC-Exo enhances cardiac repair via similar mechanism as MSCs, by promoting cardiomyocyte proliferation, reducing apoptosis, and inhibiting fibrosis. The similar miRNA profile between MSC-Exo and MSCs suggests that MSC-Exo could replace MSCs to treat myocardial infarction. The study therefore identifies MSC-Exo as a novel therapeutic strategy in the improvement of cardiac function after myocardial infarction.

## Supplementary Material

In the supplementary material, we provide evidence that MSCs derived exosomes were able to promote proliferation of H9C2 cells, inhibit apoptosis and fibrosis, and reduce infarct size.
