# Supplementary material for: MiRNA-Sequence Indicates That Mesenchymal Stem Cells and Exosomes Have Similar Mechanism to Enhance Cardiac Repair
Source: Biomed Res Int. 2017 Jan 22;2017:4150705. doi: 10.1155/2017/4150705 (PMC5292186; doi:10.1155/2017/4150705)
Supplement: Supplementary file 1 — In the supplementary material, we provide evidence that MSCs derived exosomes were able to promote proliferation of H9C2 cells, inhibit apoptosis and fibrosis, and reduce infarct size. [file 4150705.f1.pdf]

**Supplemental File**

MiRNA-sequence Indicates that Mesenchymal Stem Cells and Exosomes have Similar Mechanism to Enhance Cardiac Repair

Lianbo Shao,* Yu Zhang,* Beibei Lan, Juanjuan Wang, Zhiwei Zhang, Lulu Zhang, Pengli Xiao, Qingyou Meng, Yong-jian Geng, Xi-yong Yu, Yangxin Li

## Materials and methods

*Exosome labeling with PKH26*

Purified exosomes were labeled using a PKH26 red fluorescent labeling kit (Sigma-Aldrich) per manufacturer's instructions. The labeled exosomes were stained with PKH26 dye in 400 μL Diluent C fluid for 4 min at room temperature. An equal volume of exosome-depleted serum was added to terminate the labeling reaction. Exosomes were washed three times using 100-kDa Amicon Ultra-4 (Millipore) to remove excess dye and exosomes were repurified using isolation kit.

H9C2 and BJ cells were obtained from American Type Culture Collection (Rockville, MD), and cells were cultured in DMEM high glucose and α-MEM medium, respectively, supplemented with 10% exosome-depleted FBS, 100 U/mL penicillin, and 100 μg/mL streptomycin. The H9C2 cells and BJ cells were incubated with 100 μg/mL labeled MSC-Exo for 12 h in a 24 well plate at 37 ℃ and then washed with phosphate-buffered saline (PBS). Cells were fixed by 4% paraformaldehyde and permeabilized by 0.5% Trition-100. Nuclei were stained with DAPI (Beyotime). Uptake of labeled exosomes by H9C2 and BJ cells were analyzed using an inverted fluorescence microscope (IX51, OLYMPUS).

*Cell proliferation assay*

The effects of MSC-Exo on H9C2 cell proliferation were determined by the Edu

assay. Briefly, H9C2 cells were seeded at $5\times10^3$ cells per well in 96-well plates. Different doses of MSC-Exo (0, 100, 200, 400 μg/mL) were added to the culture medium, and then H9C2 cells were cocultured with exosomes for 48 hours. After 48 hours, the culture medium was replaced by new medium containing 30 μM Edu, and cultured for another 2 hours. Cell proliferation was measured by Edu Cell Proliferation Assay kit (Riobio) according to manufacturer's instructions.

*Apoptosis assay*

H9C2 is a clonal cell line derived from embryonic rat hearts that retains multiple cardiomyocyte phenotypes. The cells were cultured in Dulbecco's modified Eagle's medium (DMEM)- high glucose, supplemented with 10% fetal bovine serum, 100 U/mL penicillin and 100 μg/mL streptomycin. The cells were incubated with different amount of MSC-Exo (0, 100, 200, 400 μg/mL) in a humidified incubator containing 95% air and 5% $CO_2$ at 37 ℃ for 48h, and then treated with 200 μM $H_2O_2$ for 12h. $H_2O_2$-treated cells were trypsinized, washed with PBS and then pelleted by centrifugation at 200 g for 10 min. Apoptosis rate was detected by FITC Annexin V Apoptosis Detection Kit I (BD). Stained cells were analyzed by flow cytometry (Millipore).

*Masson trichrome staining*

Rats were sacrificed 1 week after myocardial infarction. Harvested hearts were arrested in diastole with 3 M KCl, washed with PBS and fixed with 4% paraformaldehyde solution for 24 h at room temperature. The hearts were simply trimmed to eliminate the upper part of ligation.

For histology, the remainder of the hearts were embedded in paraffin and cut into 5 μm thin sections. The tissues were stained by Masson trichrome staining (G1340

Masson Trichrome Stain Kit, Solarbio) to elucidate the severity of myocardial fibrosis. The average ratio of the fibrosis area to the entire LV cross-sectional area (fibrosis area %) and the average ratio of fibrosis length to entire internal LV circumference (fibrosis length %) were measured using Image J software.

*Immunofluorescence staining*

For immunofluorescence staining, the remainder of the heart was soaked in 20% sucrose solution for 4 h, embedded in OCT and kept in -80℃freezer. The frozen tissue section was cut into about 5 μm. To detect inflammation within the infarct tissues, the sections were incubated overnight with mouse anti-CD68 antibody (1:200, ab31630, Abcam), and then stained with goat anti-mouse IgG-FITC (1:200, sc2010, SANTA) at 37℃ for 1 h. Nuclei were counterstained with DAPI (200 ng/mL, Sigma). The fluorescence was detected by a fluorescence microscopy (Olympus, Japan).

*miRNA sequencing and analysis*

Total RNA was extracted from MSC and MSC-Exo using Qiagen miRNeasy Mini Kit. The sequence was detected by HiSeq 2500 (Novel Bioinformatics Co., Ltd, Shanghai, China). Fast-QC (http://www.bioinformatics.babraham.ac.uk/projects/fastqc/) software was used to assess the quality of sequencing data roundly, including quality value distribution of base, position distribution of the mass value, GC content, and so on. After quality control, RNA-seq data was mapped to miRNA, Genome and Rfam database, respectively, by MapSplice software, which was used for reads of short (<75 bp) or long sequence (>75 bp). miRNA expression was quantified by read per kilobases per millionreads (RPKM). Differentially expressed genes were defined using a FDR (False Discovery Rate) threshold and $\log_2$ FC (fold-change) analysis

through EBSeq algorithm. The threshold was defined as FDR<0.05 and $log_2$FC>1 or <-1. The target gene of miRNA was predicted by TargetScan and Miranda software. The predicted targets of the differentially expressed miRNAs were then analyzed by gene ontology (GO) categories and pathways using Fisher's exact test and $\chi^2$ test. GO analysis was performed to analyze the main function of the differential expressed genes according to the Gene Ontology data base of NCBI. Pathway annotations of genes were predicted from KEGG (http://www.genome.jp/kegg/).

**Supplemental Figure Legends**

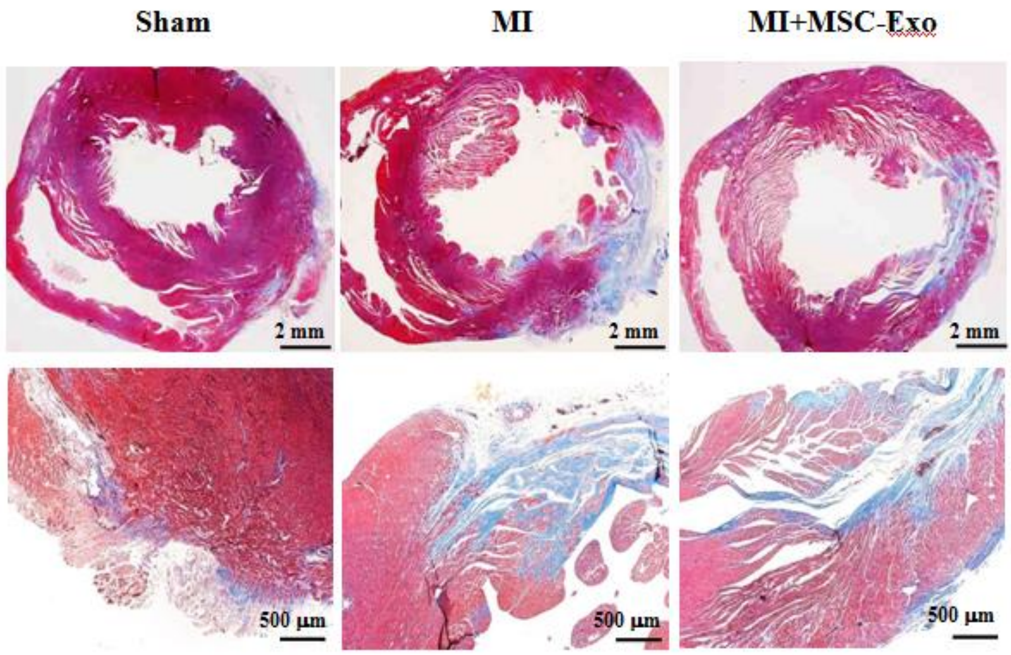


**(a)**

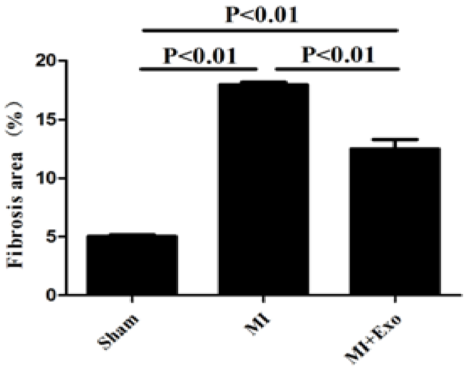


(b)

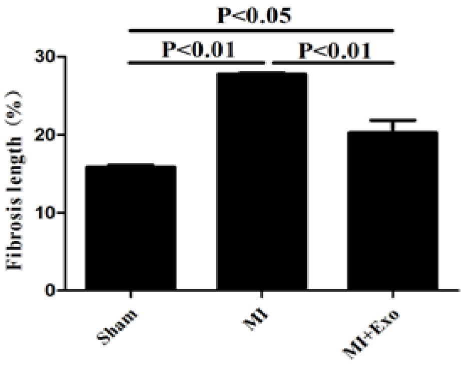


(c)

Supplemental FIGURE 1 Histological analysis for myocardial infarction (MI) sizes in each group. MSC-Exo was injected into the peri-infarct zones after MI induction. Heart samples were harvested 1 week after MSC-Exo injection. (a) Heart sections were stained with Masson trichrome: myocardium (red), scarred fibrosis (blue). The percentage of fibrotic area (b) and fibrosis length (c) was calculated and averaged (n=5/group) using Image J software.

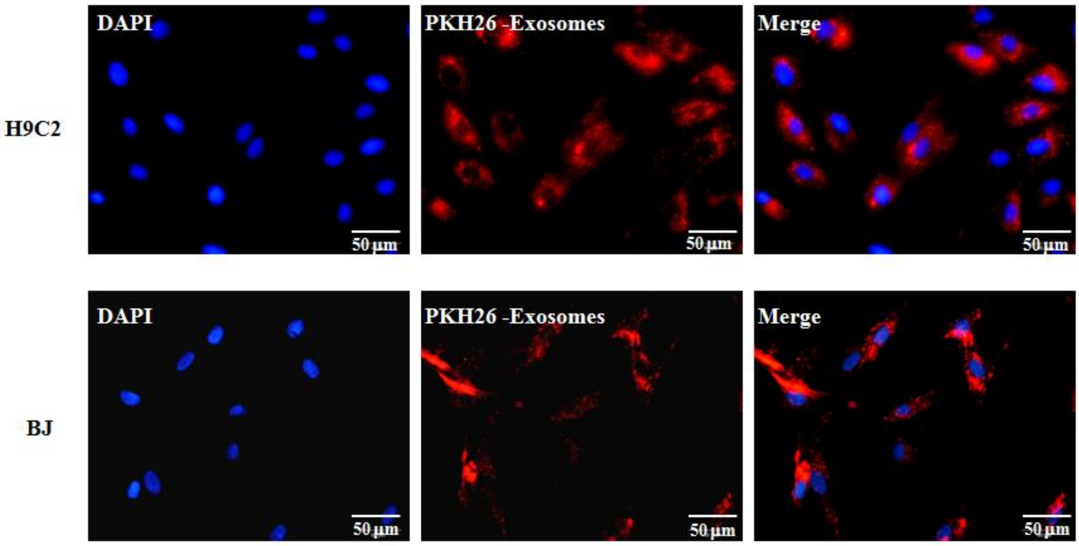


Supplemental FIGURE 2. Internalization of MSC-Exo by H9C2 and BJ cells. PKH26-labeled MSCs-Exo (red) was internalized into DAPI-labeled H9C2 and BJ cells (blue). Bar =50 µm.

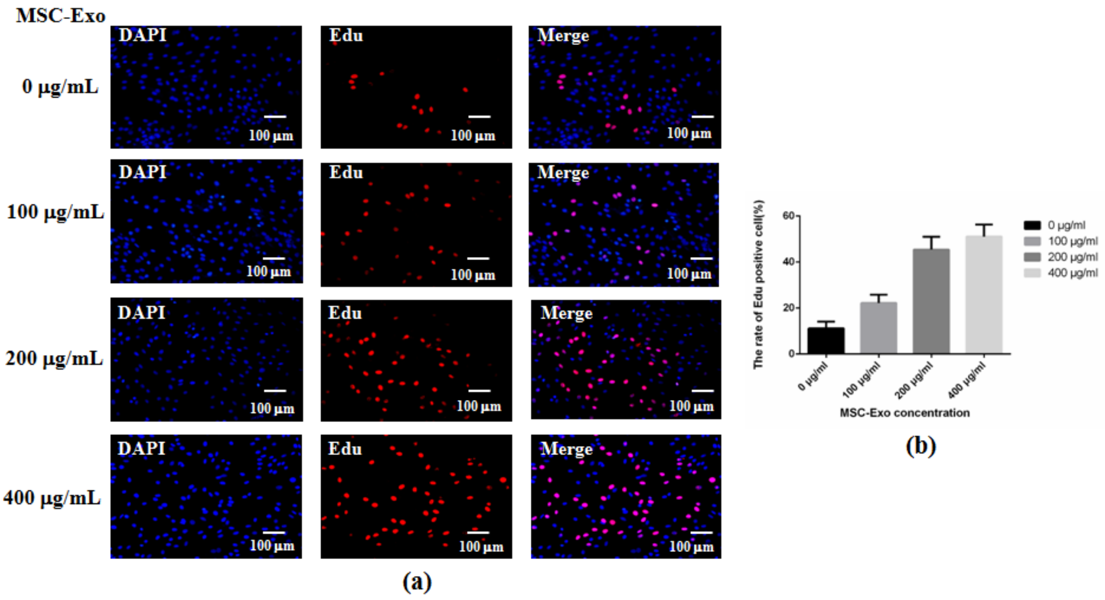


Supplemental FIGURE 3. MSC-Exo promotes proliferation of H9C2 cells in a dose-dependent manner. (a) H9C2 cells were incubated with MSC-Exo for 48 hours, and cell proliferation was measured by Edu assay. Bar =100 μm. (b) Bar graph of Edu positive cells.

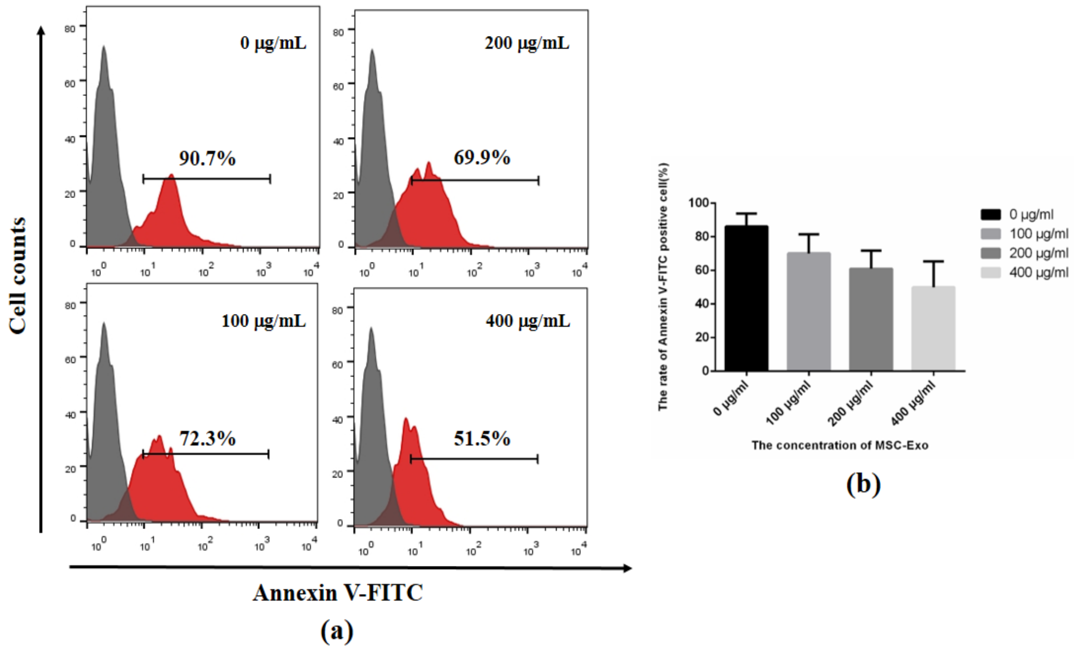


Supplemental FIGURE 4. MSC-Exo inhibits apoptosis of H9C2 cells in a dose-dependent manner. (a) H9C2 cells were incubated with MSC-Exo for 48 hours.

Apoptosis was induced by culturing cells in the presence of 200 μM $H_2O_2$ for 12 hours, and apoptotic cells were measured by flow cytometry. (b) Statistical results for Annexin V positive cells.

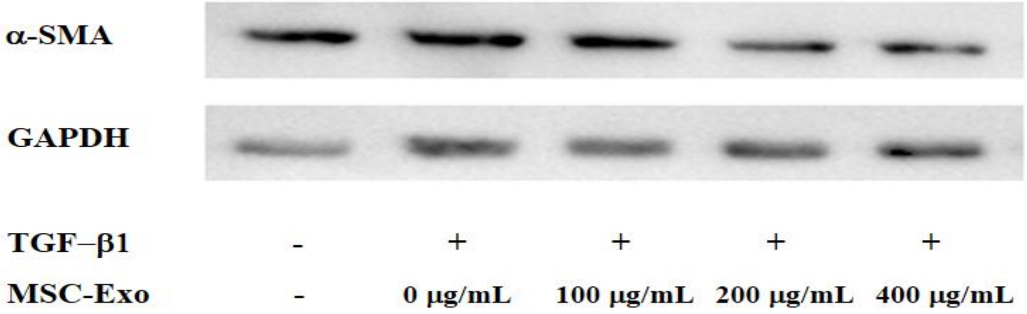


Supplemental FIGURE 5. MSC-Exo inhibits α-SMA expression in BJ cells induced by TGF-β1. BJ cells were incubated with different amount of MSC-Exo in the presence of 10 ng/mL TGF-β1. The expression of α-SMA was analyzed by Western blot.
